# Characterization of Lactobacilli Phage Endolysins and Their Functional Domains–Potential Live Biotherapeutic Testing Reagents

**DOI:** 10.3390/v15101986

**Published:** 2023-09-23

**Authors:** Robert J. Dorosky, Stephanie L. Lola, Haleigh A. Brown, Jeremy E. Schreier, Sheila M. Dreher-Lesnick, Scott Stibitz

**Affiliations:** 1 Office of Vaccines Research and Review, Division of Bacterial, Parasitic, and Allergenic Products, Center for Biologics Evaluation and Research, U.S. Food and Drug Administration, Silver Spring, MD 20993, USA; 2 Department of Marine Sciences, University of Georgia, Athens, GA 30602, USA

**Keywords:** phage endolysin, phage lysin, cell wall binding domain, enzymatic activity domain, microbial purity, *Lactobacillus*, lactobacilli

## Abstract

Phage endolysin-specific binding characteristics and killing activity support their potential use in biotechnological applications, including potency and purity testing of live biotherapeutic products (LBPs). LBPs contain live organisms, such as lactic acid bacteria (LAB), and are intended for use as drugs. Our approach uses the endolysin cell wall binding domains (CBD) for LBP potency assays and the endolysin killing activity for purity assays. CBDs of the following five lactobacilli phage lysins were characterized: CL1, Jlb1, Lj965, LL-H, and ΦJB. They exhibited different bindings to 27 LAB strains and were found to bind peptidoglycan or surface polymers. Flow cytometry based on CBD binding was used to enumerate viable counts of two strains in the mixture. CL1-lys, jlb1-lys, and ΦJB-lys and their enzymatic domains (EADs) exhibited cell wall digestive activity and lytic activity against LAB. Jlb1-EAD and ΦJB-EAD were more sensitive than their respective hololysins to buffer pH and NaCl changes. The ΦJB-EAD exhibited stronger lytic activity than ΦJB-lys, possibly due to ΦJB-CBD-mediated sequestration of ΦJB-lys by cell debris. CBD multiplex assays indicate that these proteins may be useful LBP potency reagents, and the lytic activity suggests that CL1-lys, jlb1-lys, and ΦJB-lys and their EADs are good candidates for LBP purity reagent development.

## 1. Introduction

Advances in biotechnology have provided tools to further our understanding of the interactions between humans and their microbiome and have demonstrated the importance of the microbiome in both human health and disease. This exploration of the microbiome has generated interest in using bacteria-containing products, such as over-the-counter probiotic formulations, for their purported health benefits. This also includes the investigation of live biotherapeutic products (LBPs) for the treatment or prevention of disease in controlled clinic trials. LBPs are biological products that contain live organisms, such as bacteria, are applicable to the prevention, treatment, or cure a disease or condition of human beings and are not vaccines [1,2]. LBPs can be similar to over-the-counter probiotics, but LBPs are considered different from probiotics because their intended use is as a drug. Sponsors of clinical trials investigating LBPs are generally required to perform microbiological testing to ensure participants’ safety and, in later phases, manufacturing consistency [1].

Significant challenges encountered during LBP development include the development of assays to adequately assess microbial purity (bioburden) and the potency of multi-strain LBP products. LBP microbial purity testing includes the demonstration that the final product is relatively free of contaminating microorganisms and is typically assessed using culture techniques [1]. However, it can be difficult to assess LBP microbial purity with standard culturing techniques because the growth of product organisms can obscure the detection of contaminants [3]. LBP potency is commonly a measurement of the viable cells of each strain in a product and is typically determined by standard plating methods to enumerate the colony forming units (CFU) per dose. Determining the potency of complex, multi-strain LBPs poses a challenge because it may be difficult to differentiate individual product strains based on the colony morphology or behavior on indicator media. An aim of our research program is to develop tools to improve LBP microbial purity and potency assays. Regarding microbial purity, we are interested in developing reagents that selectively eliminate or reduce product organisms and thereby allow for the subsequent outgrowth and detection of microbial contaminants by standard, unbiased plating techniques. For potency, we aim to identify the proteins that can selectively bind and identify individual product strains in the mixture by flow cytometry or by following standard plating methods. Here, we characterize the functional domains of endolysins (lysins) from phage-infecting lactic acid bacteria (LAB), specifically lactobacilli, for the future development of reagents for use in LBP potency and microbial purity assays. 

Bacteriophage (phage) lysins are peptidoglycan hydrolases produced near the end of the bacteriophage replication cycle that mediate cell lysis and the extracellular release of phage progeny [4]. Lysins from phages that infect Gram-positive bacteria exhibit a modular functional domain arrangement that typically consists of an N-terminal enzymatic domain(s) (EADs) connected by a linker region to a C-terminal cell wall binding domain(s) (CBDs) [4,5,6]. The EAD is responsible for bacterial peptidoglycan (PG) digestion and these domains can be classified by the PG bonds cleaved [7,8]. CBDs contain cell wall binding motifs, which bind specific cell wall-associated moieties, such as PG subunits, saccharides, and teichoic acids [6,8]. CBD binding is thought to be a mechanism by which the lysin EAD is targeted to the enzymatic substrate as well as a way to limit the lysin diffusion post-lysis to prevent the premature killing of nearby potential phage hosts [6,8,9]. Given their abilities to specifically bind and kill bacteria, there have been many studies investigating Gram-positive phage lysins for biotechnological applications, such as therapeutics, diagnostics, sterilizers, and bacterial detection [5,10,11,12,13,14,15]. To address the challenges associated with LBP potency and microbial purity testing, we are interested in using phage lysin CBDs to label product strains, thereby enabling the strain differentiation of individual product strains in potency assays and using the lytic activity of hololysins or EADs to reduce the number of viable product organisms to improve detection of contaminants. For CBDs to be useful potency reagents, their binding specificity should be sufficient to differentiate individual strains in a formulation so as to accurately recognize the product strains in potency assays. To be useful purity assay reagents, lysins and EADs should possess a killing activity against the product strains(s). The killing should be limited to the product organism(s) to ensure that the contaminants of concern can be detected by subsequent unbiased plating techniques. 

Lactobacilli are commonly found in the human microbiome, and they are generally considered to confer health benefits to their hosts. This is evident by the presence of lactobacilli in over-the-counter probiotic formations as well as in LBPs being investigated for therapeutic activity in controlled clinical trials (clinicaltrials.gov). A previous study from our lab used an *L. jensenii* bacterial preparation as a model LBP to demonstrate that the *L. casei* phage A2 lysin, LysA2, reduced *L. jensenii* counts and enabled the detection of contaminating (spiked in) *Escherichia coli* and *Staphylococcus aureus* [3]. Unlike the phage endolysins of pathogenic organisms, very little research has been performed on lactobacilli phage endolysins. A goal of this work is to evaluate the uncharacterized lactobacilli phage lysins for future LBP potency and purity reagent development. The major aims of this study are the following: (1) to bioinformatically identify uncharacterized lactobacilli phage lysins, (2) determine binding spectra of lactobacilli phage lysin CBDs with different predicted conserved binding domains and perform a mock potency assay, and (3) evaluate the lytic activity of lactobacilli phage lysins and EADs. Specifically, the binding characteristics of CBDs from the following five lactobacilli phage lysins: CL1, Jlb1, Lj965, LL-H, and ΦJB, which contain function domains from a variety of different classes, were determined. Notably, we provide a proof-of-concept experiment demonstrating that fluorescently tagged CBDs can be used to differentiate and assess the relative abundance of viable lactobacilli strains in the mixture. CL1-lys, jlb1-lys, and ΦJB-lys and their respective EADs were also characterized for their lytic activity. Our work demonstrates these lactobacilli phage lysins possess characteristics that support their future development as LBP microbiological testing reagents and may be suitable for other biotechnological applications. 

## 2. Materials and Methods

### 2.1. Bacterial Strains and Plasmids 

The strains and plasmids used in this study are described in Table S1. All lactobacilli were grown on De Man, Rogosa and Sharpe (MRS) agar (1.5%) under anaerobic conditions at 37 °C (Whitley workstations DG250; Microbiology International) or statically at 37 °C in MRS broth (Oxoid) under atmospheric oxygen conditions. *Escherichia coli* strains were grown in Luria–Bertani (LB) agar (1.5%) broth at 37 °C. When appropriate, *E. coli* BL21 containing expression plasmids was grown in LB medium supplemented with 100 µg/mL Ampicillin (Amp). 

### 2.2. Lysin Sequence Identification and Bioinformatic Characterization 

The lactobacilli lysins were identified in the genomes of sequenced virulent and lysogenic phages that were found in the NCBI genome database based on genomic context (i.e., downstream of tail-encoding and holin genes). The lysins were identified in the genomes of *L. paracasei* prophage CL1 (accession: KR905066.1), *L. gasseri* prophage Jlb1 (accession: KF767351), *L. johnsonii* prophage Lj965 (accession: AY459535.1), *L. delbrueckii* virulent phage LL-h (accession: EF455602.1), and *L. delbrueckii* prophage ΦJB (accession: KF188409.1). The accession numbers for each lysin are as follows: CL1, ALJ97737.1; Jlb1, AHB79914.1; Lj965, AAK27917.2; LL-h, AAC00557.1; and ΦJB, AGW43638.1. The functional domains were identified by analyzing the lysin amino acid sequences with NCBI conserved domain database [16] and HHpred [17] using standard search settings. 

### 2.3. Cloning of Full-Length Lysins, Cell Wall Binding Domains, and Enzymatic Domains 

The expression plasmids encoding the full-length lysins, EADs, and CBDs were generated by GenScript USA by cloning synthetic, codon-optimized DNA fragments into appropriate vectors. The accession number for the nucleotide sequence of each construct can be found in Table S1. For full-length lysins and EAD expression constructs, each fragment included a pET22b(+) RBS sequence upstream of the lysin/EAD coding sequence and encoded a 6x HisTag at its C-terminus and was cloned into pET22b(+) using the XbaI and XhoI restriction enzyme sites. For the fluorescent protein-CBD expression constructs, each synthesized fragment comprised the CBD coding sequences together with upstream linker region and a downstream 6x HisTag. These fragments were cloned into pSDL221 downstream of and in frame with a turboRFP CDS [18] using the SacI and XhoI restriction enzyme sites. pSDL221 was created by cloning a turboRFP CDS fragment into pET22b(+) using XbaI and SacI restriction enzyme sites. pLL-H-CBD-turboGFP was generated by cloning the CDS of turboGFP [19] into pLL-H-CBD-turboRFP upstream and in frame with the LL-H-CBD coding sequence using the XbaI and SacI restriction enzyme sites, thus replacing the turboRFP coding sequence with turboGFP. Similarly, pJlb1-CBD-TagRFP was generated by cloning a synthesized DNA fragment encoding the Tag-RFP CDS into pJlb1-CBD-turbo-RFP using XbaI and SacI. 

### 2.4. Protein Purification

Plasmid-bearing *E. coli* BL21 (DE3) strains were grown overnight at 37 °C with shaking (200 rpm) in Luria–Bertani (LB) media supplemented with appropriate antibiotics. Samples were sub-cultured the following morning in fresh LB medium and grown at 37 °C to mid-log phase, at which point expression was induced by addition of IPTG to a final concentration of 1 mM and incubated for 18 h at 16 °C with agitation. Cells were harvested by centrifugation at 4300× *g* and the pellets were stored at −20 °C until use.

Proteins were purified using a protocol adapted from the Talon Superflow manufacturer’s instructions (GE Healthcare). Fluorescent protein (FP)-CBD cell pellets were resuspended in binding buffer (50 mM phosphate buffer, 150 mM NaCl, 10 mM imidazole, pH 7.5) supplemented with Bacterial Protein Extraction Reagent (ThermoFisher), DNase (New England Biolabs), and 0.5 mg/mL Lysozyme (Invitrogen). Full-length lysin and EAD-only cell pellets were resuspended in PBS binding buffer (pH 7.2) supplemented with the same components as described above for CBD pellets. The suspended pellets were sonicated (6 × 10 sec pulses) on ice, centrifuged, and the supernatant was passed through a Millex 0.45 µM filter (Millipore). Proteins were purified from the cell-free supernatant using TALON Superflow resin (GE Healthcare) that was prepared according to the manufacturer’s instructions. Equilibrated TALON resin was incubated with the filter-sterilized supernatants for 20 min. at 4 °C with gentle shaking and then added to a gravity flow column. Resin with FP-CBD proteins was washed with phosphate buffer with 25 mM imidazole and resin with hololysins and EADs was washed with PBS with 25 mM (CL1) or 15 mM imidazole (jlb1 and ΦJB). FP-CBD proteins were eluted in phosphate buffer supplemented with 150 mM imidazole, while hololysins and EADs were eluted in PBS supplemented with 200 mM imidazole. The total protein concentration was determined using a BCA Protein Assay Kit (Pierce). Preparations were buffer exchanged against phosphate buffer supplemented with 100 mM NaCl and 15% glycerol (FP-CBDs), PBS with 15% glycerol (CL1-lys, Jlb1-lys, and EADs), or phosphate buffer pH 7 with 15% glycerol (ΦJB-lys and EAD) using Amicon ultra centrifugal filters (Millipore). Protein preparations were analyzed for purity by SDS-PAGE (Figure S1).

### 2.5. Flow Cytometry Binding Assays 

The ability of the turboRFP-CBDs to bind 27 lactic acid bacteria (Table S1) was assessed by flow cytometry as follows. Bacteria were grown overnight at 37 °C in 5 mL MRS broth under anaerobic conditions or statically under atmospheric conditions. The cells were then pelleted by centrifugation (4300× *g*, 10 min), washed twice in PBS (pH 7.2), and adjusted to OD_600_ 0.4. An aliquot of the cell suspension was mixed with purified turboRFP-CBD fusion protein preparation to a final concentration of 50 µg/mL and incubated for 20 min. at 37°C. For multiplex experiments, *L. rhamnosus* and *L. crispatus* preparations were mixed and incubated with tagRFP-jlb1-CBD (25 µg/mL) and turboGFP-LL-H-CBD (25µg/mL) and incubated at 37 °C for 20 min. After incubation, the mixtures were pelleted by centrifugation (4300 × *g*, 15 min), and washed 3X with PBS. For the CBD binding screen, the washed preparations were analyzed on a BD LSR Fortessa X-20 Flow Cytometer using a 0.5 µl/sec sample flow rate and at least 10,000 events were acquired for each sample. Cells bound by turboRFP-CBD or tagRFP-CBD proteins were detected using RFP filter settings. Data analysis was performed using FlowJo Software (FlowJo). For the multiplex mixtures, Sytox Blue Live/Dead stain (Invitrogen ThermoFisher) was added to the sample according to manufacturers’ instructions and incubated at room temperature for 20 min prior to flow cytometry analysis. The multiplex preparations were analyzed with a Cytek Aurora because this cytometer can determine counts per unit volume of mixture. The samples were analyzed using a 0.5 µl/sec flow rate and at least 20,000 events were recorded. Multiplexing data analysis was performed using SpectroFlo (Cytek). To confirm multiplex flow cytometry viable count results, mixtures were serially diluted, plated onto MRS agar, and incubated at 37 °C for 72 h. The strains used in this assay exhibit different colony morphologies, allowing for visual enumeration of each strain’s CFU.

### 2.6. Autoclaved Cell Digestion Plate Assay

The cell wall digestion and diffusion assay was performed as described previously, with modification [9]. Overnight cultures were pelleted by centrifugation, washed twice in PBS, and adjusted to an OD_600_ of 1.0. agar powder was added to the preparation (final concentration 0.7% *w*/*v*), which was autoclaved at 121 °C for 15 min. For the diffusion assay, 1 mL of the solution was added to each well of a 6-well plate (Greiner) and allowed to solidify at room temperature. For the digestion plate screen, 15 mL of the solution was added to a petri dish and allowed to solidify at room temperature. The plates were either used immediately or stored, wrapped, at 4 °C for up to two weeks. Finally, 10 µl of protein preparation (2.5 µM) or control was spotted at the center of the well and plates were incubated at 37 °C. The presence of a zone of digestion (clearing) was measured at 16 h after spotting. 

### 2.7. Turbidity Reduction Assay

The bacteriolytic activity of lysins and EADs was assessed using a turbidity reduction assay in a 96-well plate (Corning) format as previously described [20]. Briefly, bacteria were grown overnight, sub-cultured in fresh MRS broth and incubated 16 h. Cells were pelleted by centrifugation (4300× *g*, 10 min, 4°C), and washed two times in an equal volume of PBS. The OD_600_ was assessed prior to the third wash step and cells were suspended in an appropriate buffer. A 100 µl aliquot of the cell suspension and a 100 µl of lysin or EAD solution of pre-determined concentration was added to each well such that the resulting starting OD_600_ was 0.9 (± 0.1) for biochemical characterization and 0.6 (± 0.1) for lytic spectrum. Controls for each strain consisted of a mixture of only bacteria and buffer. After addition of lysins, the plates were immediately placed into a Cytation 3 Imaging Reader (Biotek Instruments Inc) set at 37 °C with continuous shaking and OD_600_ was measured every minute for 1 h using the Gen5 v2.06 program (Biotek). The lytic activity was calculated after 60 min as previously described: [∆OD_600_ endolysin treated − ∆OD_600_ control (buffer only)]/initial OD_600_ [21]. Following the assay, CFUs were determined from samples taken from each well after serial dilution and plating on MRS agar plates that were incubated under anaerobic conditions at 37 °C for 48 h. 

### 2.8. Effect of pH and NaCl on Lysin and EAD Activity

Turbidity reduction assays were used to determine the optimal lytic conditions for each lysin and EAD. To determine the effect of pH on enzymatic activity, hololysins or EAD (1.0 µM) were added to *L. delbrueckii* cells suspended in the following buffers: 50 mM sodium acetate for pH 4.5 and 5.0, or 50 mM phosphate buffer for pHs 5.8–8.0. To study the effects of NaCl concentration on hololysin and EAD cytolytic activity, hololysins or EAD (1 µM) were added to *L. delbrueckii* cells that were suspended in 50 mM phosphate buffer pH 5.8 supplemented with increasing concentrations of NaCl (0–200 mM).

## 3. Results

### 3.1. Lactobacilli Phage Lysin CBDs Bind The Surface of a Variety of Lactobacilli 

**Figure 1:**
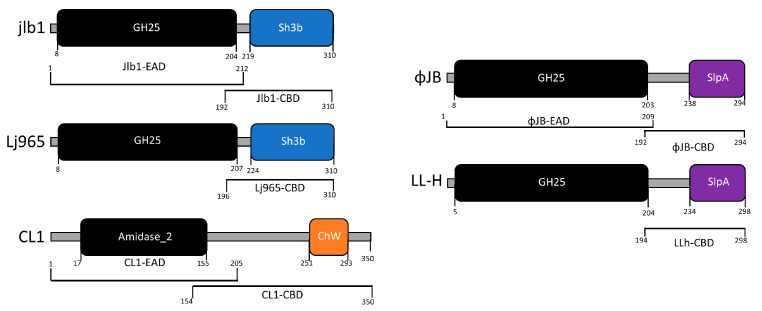
Domain architecture of lysins characterized in this study. Lysins were identified in the genomes of sequenced virulent and lysogenic phages that were found in the NCBI genome database. The functional domains were identified by analyzing the amino acid sequences against NCBI conserved domain database and by HHpred using standard search settings. The EADs identified, amidase-2 (IPR002502) and glycoside hydrolase family 25 (IPR018077), are shown in black. EADs are connected to their respective CBDs by linker domains (gray bars). CBD domains were labeled according to their family: ChW (orange), Sh3b (blue), SlpA (purple). The brackets below each schematic indicate the amino acid sequence for each EAD and CBD characterized in this study.

**Figure 2:**
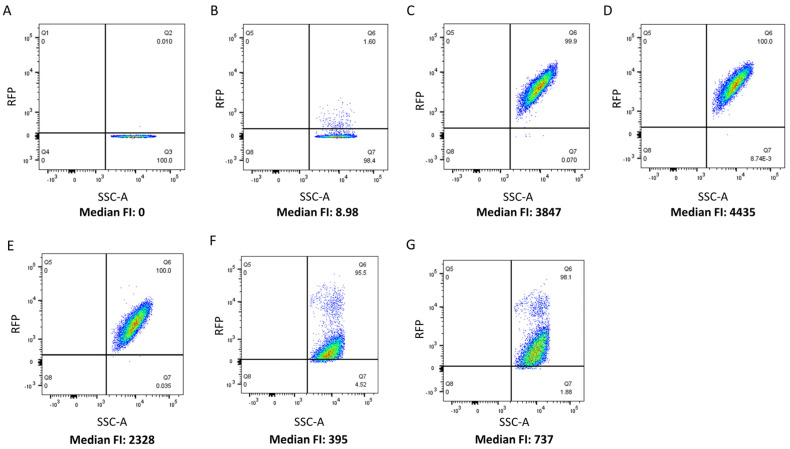
Flow cytometry analysis of TurboRFP-CBD binding. Flow cytometry dot plots relating counts (SSC-A) to red fluorescence intensity. *L. gasseri* ATCC 9857 was pre-incubated with (**A**) PBS, (**B**) TurboRFP, (**C**) TurboRFP-CL1-CBD, (**D**) TurboRFP-Lj965-CBD, (**E**) TurboRFP-jlb1-CBD, (**F**) TurboRFP-LL-H-CBD, and (**G**) TurboRFP-ΦJB-CBD. Representative binding data from two independent experiments are shown.

The CBD cell binding capability of 5 LAB phage lysin CBDs, shown schematically in Figure 1, was assessed against 27 LAB strains using flow cytometry and protein fusions of the CBDs to a red fluorescence protein. The CBDs from *L. paracasei* prophage CL1, *L. gasseri* prophage Jlb1, *L. johnsonii* prophage Lj965, *L. delbrueckii* virulent phage LL-h, and *L. delbrueckii* prophage ΦJB lysins were chosen for analysis because they possess functional binding domains that have previously been bioinformatically identified in the lactobacilli phage [6] and they have not be experimentally characterized. A positive red fluorescence signal shift compared to the PBS and TurboRFP controls, was indicative of CBD binding (Figure 2). For example, a positive red fluorescence signal was observed when *L. gasseri* 9857 was mixed with TurboRFP-CL1-CBD, TurboRFP-Lj965-CBD, TurboRFP-Jlb1-CBD, TurboRFP-LL-H-CBD, or TurboRFP-ΦJB-CBD (Figure 2C–G). It is important to note that the median fluorescence intensity and dot plot foci shape varied with the CBDs tested, suggesting that the different CBDs may exhibit different extents, affinities, and patterns of binding.

The bank of LAB strains tested comprises 25 lactobacilli strains, a *Lactococcus lactis* strain, and an *Enterococcus durans* strain (Table 1). The CBDs from phages CL1 (ChW), Lj965 (SH3b), and jlb1 (SH3b) bound 63%, 78%, and 70% of the strains tested, respectively. These CBDs bound strains of their respective host species (e.g., CL1-CBD and *L. casei* 393) as well as the *Enterococcus* and *Lactococcus* strains tested (Table 1). The CBDs from *L. delbrueckii* phages ΦJB and LL-H, both with SlpA functional binding domains, bound 52% of the strains tested. These two CBDs share 71% amino acid identity with each other, and both bound to the same subset of the lactobacilli strains tested. The binding data revealed differences in the scope of strains bound by the five CBDs tested and also showed that the CBDs exhibited strain-level binding differences suggesting that they may be useful tools to discriminate lactobacilli in a mixture as well as in other biotechnological applications requiring specific labelling.

### 3.2. Cell Wall Constituents Differentially Influence CBD Cell Surface Binding

**Figure 3:**
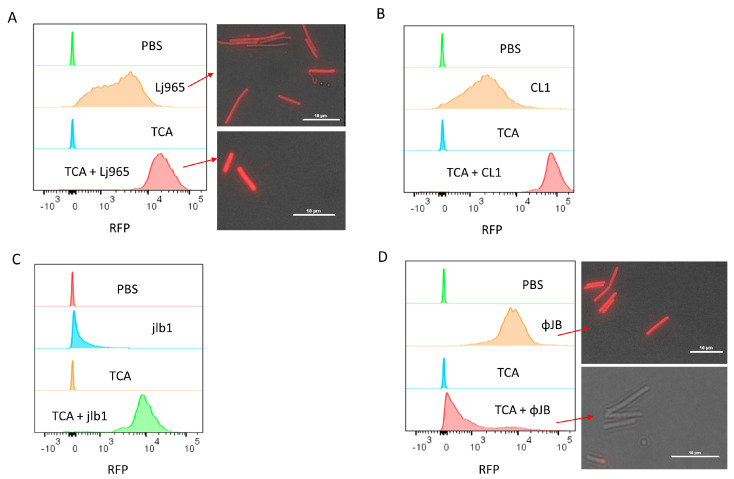
Flow cytometry and fluorescence microscopy of TurboRFP-CBD binding to TCA-treated calls. Cells were resuspended in 1 mL PBS or PBS 10% TCA and heated for 15 min at 98 °C. After heat treatment, the cells were washed four times by centrifugation (4300× *g*, 10 min) and resuspended in PBS. Panels show overlaid histograms of cells pre-treated with PBS or TCA and then incubated with PBS (control) or 50 µg/mL of (**A**) TurboRFP-Lj965-CBD-treated *L. delbreuckii*, (**B**) TurboRFP-CL1-CBD-treated *L. delbrueckii*, (**C**) TurboRFP-jlb1-CBD-treated *L. crispatus*, or (**D**) TurboRFP-ΦJB-CBD-treated *L. delbreuckii*. Representative data from two independent experiments are shown. The red arrows in A and D point to the respective samples visualized using a Nikon eclipse Ci fluorescent microscope equipped with an AT-TRITC filter. Image analysis was conducted with NIS Elements BR software and scale bars = 10µm.

The flow cytometry binding data showed that fluorescence intensity varied when the same strain was incubated with different CBDs or when the same CBD was incubated with different strains (Figure 2 and Figure 3). To better understand the nature of CBD-specific ligands, cells were pre-treated with trichloroacetic acid (TCA) prior to flow cytometry binding assays. TCA is commonly used for the purification of peptidoglycan and is known to remove PG-associated polymers, such as carbohydrates like (lipo)teichoic acids [22,23,24]. As shown in Figure 3A, pre-treatment with TCA resulted in an approximately 8-fold increase in the fluorescence intensity of Lj965-CBD bound *L. delbrueckii* cells relative to the untreated cells, with the TCA-treated cells also appearing significantly brighter under fluorescence microscopy after incubation with the same RFP-CBD preparation. A similar effect of TCA pre-treatment on fluorescence intensity was observed with CL1-CBD and jlb1-CBD binding to *L. delbrueckii* and *L. crispatus* 33197, respectively (Figure 3 B,C). The finding that the removal of cell surface constituents improved the binding of these lysin CBDs, representing the SH3b and CHW domains, is consistent with these CBDs binding either PG or a PG subunit. Additionally, CL1-CBD is similar to a *L. casei* lysin CBD previously shown to bind to an amidated PG cross-bridge common in a diversity of LAB [25]. In contrast, the fluorescence intensity of ΦJB-CBD (SlpA)-bound TCA pre-treated *L. delbrueckii* cells was much lower than the PBS-treated cells (Figure 3 D). These cells were also no longer fluorescent under fluorescence microscopy, suggesting that the TCA pre-treatment reduced or removed the ΦJB-CBD ligand from the cell surface (Figure 3D). TCA-mediated removal of the ΦJB ligand suggests that the ligand is possibly associated with PG but is not PG itself. It is of note that ΦJB-CBD contains a SlpA binding domain that is similar to the *Lactobacillus* S-layer protein cell wall binding domains that anchor the S-layer to the cell surface by binding to lipoteichoic acids [26,27]. 

### 3.3. Fluorescently Labeled CBDs Can Be Used to Differentiate and Enumerate Viable L. crispatus and L. rhamnosus in Mixture by Flow Cytometry

**Figure 4:**
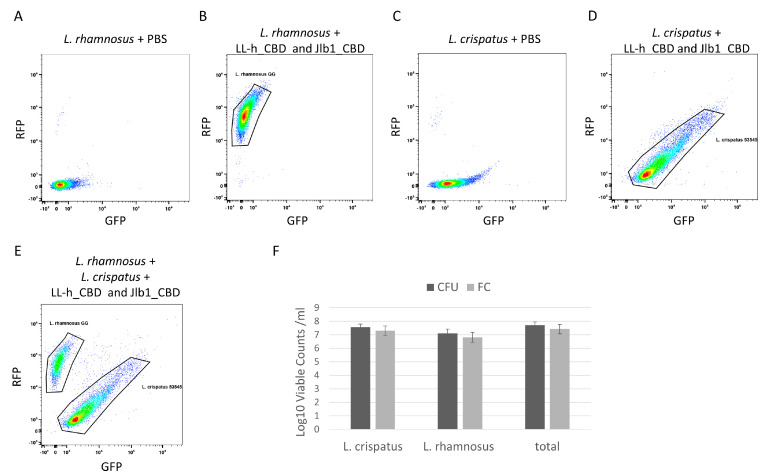
Flow cytometry analysis of *L. crispatus* 53545 and *L. rhamnosus* GG strain mixture. Dot plot diagrams flow cytometric analyses relating GFP and RFP intensity of strains or strain mixtures as follows: (**A**) *L. rhamnosus* GG PBS control, (**B**) *L. rhamnosus GG* incubated with TagRFP-jlb1-CBD, TurboGFP-LL-H-CBD (25µg/mL), and Sytox Blue Live/Dead (**C**) *L. crispatus* 53545 PBS control, (**D**) *L. crispatus* 53545 incubated with TagRFP-jlb1-CBD, TurboGFP-LL-H-CBD (25µg/mL), and Sytox Blue Live/DeI (**E**) strain mixture incubated with TagRFP-jlb1-CBD, TurboGFP-LL-H-CBD (25 µg/mL), and Sytox Blue. The panel in (**F**) shows a comparison of viable counts determined by FC to those of the same samples by plating and CFU counting. Data points represent the mean ± standard deviation from six biological replicates in two independent experiments. A t-test was used to compare viable counts obtained by FC and CFU; ns, nonsignificant (*p* > 0.05).

Flow cytometry was used to enumerate viable *L. crispatus* and *L. rhamnosus* in a mixture by the specific binding of fluorescently labeled CBDs. A mixture of *L. crispatus* 53545 and *L. rhamnosus* GG was prepared, and a live/dead stain was added to allow counting of live cells only. Single-strain preparations of *L. crispatus* and *L. rhamnosus* were incubated with both TagRFP- jlb1-CBD and turboGFP-LL-H-CBD and were analyzed by flow cytometry. Single-strain controls exhibited distinct foci that were used to define the gates applied to enumerate the two strains in the mixture (Figure 4B,D). Flow cytometry dot plots of the two-strain cell mixture have two distinct density foci, each corresponding to the pattern observed in the *L. crispatus* 53545 or *L. rhamnosus* GG single-strain controls (Figure 4E). The flow cytometer used in these experiments measures the sample volume analyzed, which, in combination with the fluorescent CBDs and a viability stain (Figure S2), enables the enumeration of viable cells (VC) of each strain per unit volume. The flow cytometry analysis of the strain mixture detected 4.3 × 10^7^ VC/mL (SD = 1.3 × 10^7^ VC/mL) viable *L. crispatus,* 3.83 × 10^7^ VC/mL (SD = 3.43 × 10^6^ VC/mL) viable *L. rhamnosus,* and 8.1 × 10^7^ VC/mL (SD = 1.5 × 10^7^ VC/mL) total viable cells. To confirm the flow cytometry results, the mixtures were serially diluted and plated on MRS agar to allow the CFU determination for each strain based on differences in colony morphology. The viable counts of each strain and of the total mixture, as measured by flow cytometry and CFU, were not statistically different (Figure 4F). This suggests that the fluorescent CBD flow cytometry analysis can accurately enumerate the viable cells of each strain in a mixture. These results support the future development of lysin CBDs as reagents to enable differentiation of strains in LBP potency testing and warrant further investigation with lyophilized LBP preparations to determine if the product matrix interferes with CBD binding. 

### 3.4. Phage CL1, jlb1, and ΦJB Hololysins and EADs Exhibit Broad Cell Digestion Activity

**Figure 5:**
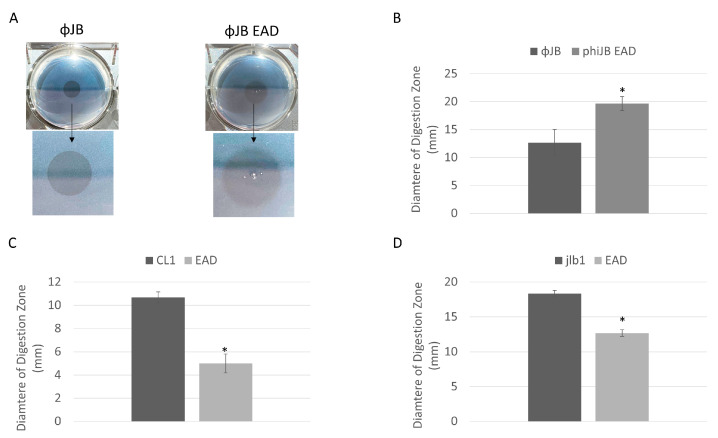
Lysin and EAD cell digestion activity. (**A**) Digestion clearing zone formed on autoclaved *L. delbrueckii* 15808 cells spotted with 10 µM of ΦJB-lys (**left**) and ΦJB-EAD (**right**) and incubated at 37 °C for 1 h. The arrow points to a zoomed-in image of the digestion zone to better show the border characteristics. (**B**–**D**) Diameters of the digestion zones formed after spotting 10 µM of (**B**) ΦJB-lys and ΦJB-EAD on autoclaved *L. delbrueckii* 15808 and 10 µM of (**C**) CL1-lys and CL1-EAD and (**D**) Jlb1-lys and Jlb1-EAD on autoclaved *L. jensenii* SJ-7A-US. Representative data from three independent experiments are shown (*n* = 3). A t-test was used to compare zones formed by the full lysin and EAD domain; *, significant (*p* < 0.05).

The endolysin digestive activity was assessed by spotting purified CL1-lys, Jlb1-lys, ΦJB, or their isolated EADs onto agar (0.7%) supplemented with autoclaved LAB cells. Digestive activity was indicated by the presence of a clearing zone where the preparations were spotted (Figure 5A). The LAB strains chosen included, for each lysin, a strain that bound the CBD of the lysin and one that did not. The lysin digestion assay shows that the EAD and full-length CL1, Jlb1, and ΦJB lysins exhibit digestive activity on the lactobacilli strains tested (Table 2). Interestingly, the digestive activity of each lysin and EAD was observed for some strains previously found not to be bound by the respective CBDs (e.g., *L. plantarum* 8014 for all hololysins and EADs), suggesting that CBDs are not required for the digestive activity of these lysins. Interestingly, the diameters of the zones of digestion from spotting the full-length lysin and EAD were different. The diameter of the digestion zone formed on *L. delbrueckii* 15808 by ΦJB-lys was significantly smaller than that formed by ΦJB-EAD (Figure 5A,B). Additionally, the border of the zone of digestion formed by ΦJB-lys was crisp and well-defined, while that formed by ΦJB-EAD was diffuse (Figure 5A). In contrast, the digestion zone diameter that developed on *L. jensenii* SJ-7A-US autoclaved cells spotted with CL1-lys and jlb1-lys were significantly larger than those that formed by CL1-EAD and jlb1-EAD (Figure 5C,D). These data indicate that the jlb1, CL1, and ΦJB lysin CBDs are not absolutely required for cell wall digestion but may influence digestive activity and diffusion of the lysins. The CL1-lys and Jlb1-lys CBDs are required for full digestion activity in this assay, while ΦJB-lys activity was improved by the removal of the CBD. The lower activity observed for ΦJB-lys compared to ΦJB-EAD may be due to sequestration of ΦJB-lys to cell wall debris by CBD binding. Alternatively, the lower mass of the EAD may lead to a higher rate of diffusion, explaining the larger digestion zone. However, increased diffusion was not observed with CL1 and jlb1 lysins, and this does not explain the observed difference in the crisp versus diffuse zone borders.

### 3.5. Characterization of Hololysin and EAD Lytic Activity

**Figure 6:**
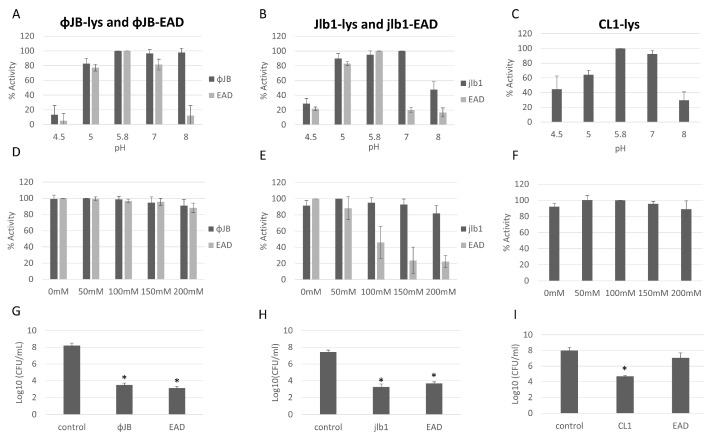
Optimization of conditions for hololysin and EAD activity. The effects of pH (**A**–**C**) and NaCl concentration (**D**–**F**) on lysin and EAD activity against *L. delbrueckii* 15808. The values for each parameter are presented as a percentage of the lytic activity in relation to the highest activity observed. (**G**,**H**) Reduction in *L. delbrueckii* 15808 viable counts observed after incubation with 1 µM ΦJB-lys and ΦJB-EAD (**G**), Jlb1-lys and Jlb1-EAD, and with CL1-lys and CL1-EAD in phosphate buffer pH 5.8 for 1 h (**I**). Bars show the mean ± standard deviation from two independent experiments (*n* = 6). A t-test was used to compare the CFU obtained after lysin or EAD exposure to the control; *, significant (*p* < 0.05).

The antibacterial activity and optimal buffer conditions of the lysins and EADs were investigated by turbidity reduction assays with *L. delbrueckii* 15808. Viable *L. delbrueckii* 15808 was chosen for these experiments because the lysins and EADs tested exhibited potent lytic activity against this strain. The optimal buffer pH and NaCl concentrations were determined by testing the lytic activity across a range of pH (4.5–8) and NaCl (0–200 mM) concentrations. ΦJB-lys exhibited high lytic activity (80–100%) at pH 5, 5.8, 7, and 8, with activity dropping sharply to 16% at pH 4.5 (Figure 6A). ΦJB-EAD activity remained high in pH 5, 5.8, and 7.0 and dropped dramatically to below at pH 4.5 and 8.0 (Figure 6A). Jlb1-lys exhibited high lytic activity (>80%) at pH 5, 5.8, and 7 and activity was reduced at pHs outside of that range (Figure 6B). Similarly, Jlb1-EAD showed high lytic activity (80–100%) at pH 5 and 5.8 and the activity dropped steeply outside of that range (Figure 6B). CL1-lys exhibited high lytic activity (80–100%) at pH 5.8 and 7 and retained approximately 60% activity at pH 5, although CL1-lys activity dropped to below 50% at pHs 4.5 and 8 (Figure 6C). CL1-EAD exhibited marginal lytic activity against *L. delbrueckii* and all other strains tested, and pH and NaCl optimization was not performed. These data show that these lysins and EADs retained relatively high lytic activity at pH 5.8–7. ΦJB-EAD exhibited activity similar to the full-length lysin in the buffers at pH 4.5, 5.0, 5.8, and 7.0, but showed less activity in the pH 8.0 buffer. Similarly, Jlb1-EAD had activity comparable to Jlb1-lys in the buffers at pH 4.5, 5, and 5.8, but was less active than the full-length lysin the in buffers between pH 7 and 8. These data suggest that the presence of the ΦJB and Jlb1 CBD improves the activity and/or stability of the lysin at pHs outside the optimal range. The effect of NaCl concentration on lysin and EAD activity was assessed in phosphate buffer pH 5.8. For ΦJB-lys, ΦJB-EAD, CL1-lys, and Jlb1-lys, the addition of up to 200 mM NaCl had little or no effect on lysin activity and these proteins retained high lytic activity (Figure 6D–F). However, the Jlb1-EAD was more sensitive to salt concentration at every concentration tested, with activity dropping to approximately 20% at 150 and 200 mM NaCl. Thus, similarly to what was observed for the effects of pH on activity, the Jlb1-EAD was more sensitive to deviations from optimal conditions, suggesting again that the presence of this CBD exerts a stabilizing influence on its associated EAD.

### 3.6. Phage CL1, jlb1, and ΦJB Hololysins and EADs Show Potent Antibacterial Activity and Different Lytic Spectra

The lysin and EAD killing activity was assessed by comparing viable *L. delbrueckii* 15,808 CFU/mL recovered after incubation with lysins, EADs, or a buffer control (phosphate buffer pH 5.8) for 1 h. As shown in Figure 6 G–I, incubation with ΦJB-lys, ΦJB-EAD, Jlb1-lys, Jlb1-EAD, or CL1-lys significantly reduced the *L. delbrueckii* viable counts compared to the buffer control. The reduction observed with the Jlb1-EAD and ΦJB-EAD was not significantly different than the respective full-length lysin, indicating that the CBD is not necessary for killing activity under these conditions. Interestingly, CL1-EAD alone did not display a significant reduction in CFU compared to the control (Figure 6I). These data demonstrate that ΦJB-lys, jlb1-lys, CL1-lys, ΦJB-EAD, and jlb1-EAD exhibit potent killing activity against *L. delbrueckii.*

To further investigate the lytic spectrum of each lysin and EAD, we examined their activity through turbidity reduction assays against additional strains. These strains, as shown in Table 3, are a subset of the strains included in the CBD binding screen. The strains were prepared as described in the turbidity reduction assay methods, and OD_600_ was measured for an hour after treatment with 1 µM of purified protein (hololysin or EAD) in phosphate buffer pH 5.8 (jlb1 and CL1) or pH 7.0 (ΦJB). All of the lysins and EADs tested showed some lytic activity against at least of one the strains tested, with the exception of *L. crispatus* 33197 and *Enterococcus durans* RM86, in which none of the proteins lysed in these conditions (Table 3). Jlb1-lys had a strong lytic activity against *L. gasseri* SV-16A-US, *L. jensenii* SJ-7A-US, and *L. delbrueckii* 15808, whereas Jlb1-EAD only had strong activity against *L. delbrueckii*. This suggests that the Jlb-1 CBD is required for optimum lytic activity against *L. jensenii* SJ-7A-US and *L. gasseri* SV-16A-US but is not required for optimum lytic activity of *L. delbrueckii* 15808 (Table 3). CL1-lys exhibited strong lytic activity against *L. delbrueckii* and *L. jensenii* SJ-7A-US and moderate activity against *L. gasseri* SV-16A-US. Compared to CL1-lys, CL1-EAD showed reduced lytic activity against the same strains (Table 3), indicating that the CBD is required for optimal lytic activity. ΦJB-lys and ΦJB-EAD exhibited strong lytic activity against *L. delbrueckii* 15808 and weak activity against *L. gasseri* SV-16A-US. ΦJB-EAD also showed weak activity against *L. jensenii* SJ-7A-US. These data suggest that the CBD is not required for optimal lytic activity of ΦJB. 

### 3.7. ΦJB-CBD Limits ΦJB-lys Diffusion and Reduces Lytic Activity

**Figure 7:**
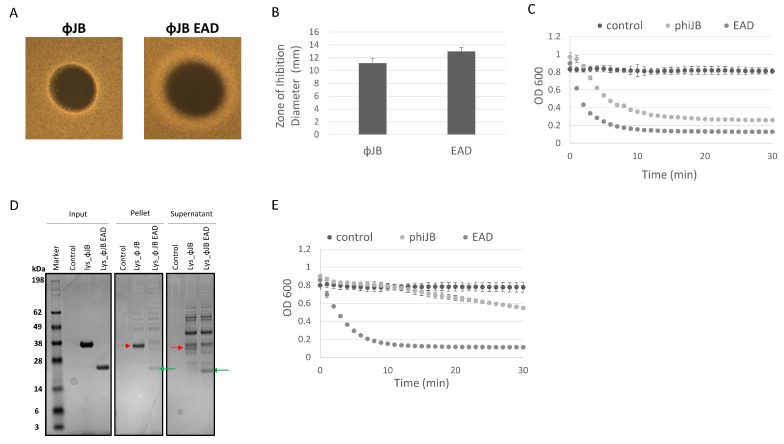
The contribution of ΦJB-CBD to ΦJB-lys lytic activity. (**A**) 5 µl of a 10 µM solution of ΦJB-lys or ΦJB-EAD was spotted onto MRS soft agar overlays (0.7%) seeded with *L. delbrueckii* 15808 and then incubated anaerobically for 16 h at 37 °C. (**B**) The diameter of the zone of inhibition was measured after 16 h of incubation. Data points (B) represent the mean ± standard deviation from two independent experiments (*n* = 6). (**C**) ΦJB-lys and ΦJB-EAD lysis of *L. delbrueckii* 15808 as measured by a turbidity reduction assay. Each protein was added to *L. delbrueckii* 15808 cells at a final concentration of 2.0 µM and OD_600_ was followed for 30 min at 37 °C. (**D**) The lysis reaction was fractionated into pellet and supernatant by centrifugation (14,000× *g*, 10 min) and samples were analyzed by SDS-PAGE and Coomasie blue staining. A representative biological replicate from two independent experiments run in triplicate is shown. The red arrow points to ΦJB-lys and the green arrow points to ΦJB-EAD. (**E**) Supernatant samples were mixed with live *L. delbrueckii* cells and the reduction in OD_600_ was monitored for 30 min. and plotted. The data points in C and E are Mean ± SD (*n* = 3).

The differences observed in the clearing zones of ΦJB-lys and ΦJB-EAD in the autoclaved cell digestion assay, as shown in Figure 5A, led us to further investigate the lytic activity of these proteins via soft agar overlays with live cells and turbidity reduction assays to investigate the location (pellet or supernatant) of the lysin and EAD in lysates by SDS-PAGE analysis. Similarly to what was observed in the autoclaved cell digestion assay, ΦJB-EAD formed larger zones of inhibition that exhibited diffuse borders when compared to ΦJB-lys when spotted on soft agar overlays seeded with live *L. delbrueckii* 15808 cells (Figure 7A,B). These data are consistent with the CBD diffusion hypothesis that ΦJB-lys exhibited a smaller killing zone with crisp boundaries because ΦJB-CBD, in the full-length lysin, binds to cellular debris, thus reducing its diffusion to, and lysis of, nearby cells. As described above, the diffusion observation may also be explained by the reduced molecular weight of ΦJB-EAD improving its ability to diffuse through the soft agar matrix. Interestingly, ΦJB-EAD-treated *L. delbrueckii* 15808 cells exhibited a faster reduction in OD_600_ and a lower final OD_600_ at 30 min than the cells incubated with the full-length lysin ΦJB-lys under the test conditions (Figure 7C). This indicates that ΦJB-EAD showed slightly stronger lytic activity than the native full-length lysin, suggesting that the presence of the CBD may tether the full lysin to cellular debris, thereby reducing its diffusion and ability to lyse cells. To test this, an *L. delbrueckii* 15808 lysis reaction was performed with both proteins, the lysis reaction was fractionated by centrifugation, and the proteins in the supernatant and pellet were visualized by SDS-PAGE. As shown in Figure 7D, in the lysate formed by ΦJB-lys, the ΦJB-lys band (indicated by the red arrow) was observed in both fractions, although the band observed in the supernatant fraction was less intense than that in the pellet fraction. In the lysate formed by the action of ΦJB-EAD, the ΦJB-EAD band (indicated by the green arrow) appeared stronger in the supernatant fraction compared to the pellet (Figure 7D). These results indicate that the CBD domain mediates lysin binding to cellular debris. Moreover, ΦJB-lys exhibited a large reduction in lytic activity when the supernatant fraction was used in a subsequent lysis reaction, while no reduction was observed when the ΦJB-EAD supernatant was used. These data are consistent with the hypothesis that ΦJB-EAD may exhibit higher lytic activity than ΦJB-lys because the CBD-less EAD protein is not sequestered by binding to cellular debris. The CBD of *Clostridioides difficile* phage endolysin CD16/50L has recently been shown to anchor the full lysin to cell debris and reduce lytic activity in sequential lysis experiments [9]. In the natural context of phage infection, the ΦJB-CBD may adhere the endolysin to cellular debris to preserve uninfected cells for additional rounds of phage infection. However, in the context of an LBP purity assay, increasing diffusion and lytic activity may be beneficial.

## 4. Discussion

The bacterial killing and cell wall binding activity of phage endolysins has driven an investigation of these proteins, or their individual functional domains, for use in a broad array of biotechnological applications [5]. The specific killing and cell binding activities typical of phage endolysins make them candidates for investigation as reagents in LBP purity and potency assays, respectively. Many lactobacilli carry prophages in their genomes [28], and these prophages can be mined for endolysins and other phage-encoded proteins to be developed as tools to characterize and test LBPs. For example, virion-associated peptidoglycan hydrolases (VAPGHs) are proteins displayed on the phage virion and degrade the host PG from “without”, thereby facilitating phage genome injection [5]. Given these proteins have evolved to degrade host PG from the “without” and that our application is an exogenous application that lysis from “without”, these VAPGH also have potential as LBP purity reagents. Our previous work provided a proof-of-concept demonstrating the potential of endolysins in LBP purity assays [3]. In this study, we sought to better understand the role of lactobacilli lysin functional domains for future development as LBP microbiological testing reagents.

To further characterize and assess the potential of lactobacilli phage lysin CBDs as LBP microbiological testing reagents, five lactobacilli phage endolysin CBDs with three different conserved functional binding domains were characterized. The lysin CBDs studied here were identified in publicly available lactobacilli phage genomes and chosen because they possess binding domains that are commonly found in lactobacilli phage endolysins in bioinformatics studies but which have not been previously characterized [6]. A screen for binding to different strains was performed to establish the binding range of each CBD and the general nature of ligands bound. There have been few published studies evaluating lactobacilli phage lysin CBD binding. In two of these studies, an *L. casei* prophage lysin CBD with a ChW domain and an *L. fermentum* prophage lysin CBD with LysM domains were shown to bind peptidoglycan and to exhibit broad-spectrum LAB binding ability [22,25]. To our knowledge, the present study is the first to characterize lysin CBDs from phages CL1, Lj965, jlb1, LL-H, and ΦJB, and the first to characterize lactobacilli phage lysin CBDs comprising SH3b and SlpA domains. Our results showed that the CBDs exhibited a range of binding activities, with the ChW and SH3b CBDs generally binding a more diverse collection of LAB, including *Lactobacillus*, *Lactiplantibacillus*, *Limosilactobacillus*, *Lacticaseibacillus*, *Lactococcus*, and *Enterococcus*, and with the SlpA domain CBDs binding a narrower spectrum of lactobacilli. The CL1-CBD binding data herein conforms to a previous study on a similar *L. casei* phage lysin CBD, showing that CL1-CBD exhibits a broad LAB binding capability and that it binds peptidoglycan [25]. Similarly, our results also suggest that the SH3b binding domain CBDs, Lj965-CBD and Jlb1-CBD, bind peptidoglycan. These findings are consistent with previous studies characterizing the ligand bound by lysin CBDs harboring SH3b functional binding domains [12,23,29,30,31]. In contrast, our data suggest that the ΦJB-CBD, containing an SlpA domain, binds a molecular structure associated with the cell surface, such as lipoteichoic acid or another exopolysaccharide. While many lysin CBDs studied have been shown to bind peptidoglycan, some lysin CBDs, namely, the *C. difficile* phage endolysin CD16/50L and *Listeria monocytogenes* phage endolysins PlyP35 and Ply500, have been shown to bind exopolysaccharides and teichoic acids, respectively [9,32,33]. Moreover, the ΦJB-CBD binding domain is similar to the cell wall binding domains of *Lactobacillus* S-layer proteins, and these domains have been shown to bind lipoteichoic acids [26,27]. Future work will investigate a more precise identification of the ligands bound by ΦJB-CBD. Taken together, the binding screen and ligand data suggest that the biotechnological applicability of the lactobacilli phage lysin CBDs characterized may not be limited to LBP potency assays as they may be useful tools for surface display of proteins. Importantly, the binding data indicated differences in the CBD binding spectra, and these characteristics may be useful to differentiate strains in a mixture, an important aspect of multi-strain LBP potency assay development.

The ideal potency reagent would have enough binding specificity to differentiate strains from each other in a mixture so that the viable counts of each strain can be enumerated. The level of binding specificity required would depend on the number and diversity of product strains. For a less complex product consisting of a few diverse strains, a CBD with a broad binding ability may work, but as the product complexity increases, CBDs with narrower spectra would be more suitable. A published study evaluated the potency of a probiotic product using flow cytometry to detect and enumerate viable *Lactobacillus* and *Bifidobacterium* probiotic strains by the binding of fluorescently conjugated polyclonal antibodies [34]. The development of similar flow-cytometry based assays using phage lysin CBD-based reagents may offer some improvements in terms of developing appropriate reagents. Lysin genes and their encoded CBDs are plentiful because most lactobacilli harbor prophages containing such genes [28], and the presence of such genes in the genome of a particular strain is already an indication that the CBD is likely to bind to that strain. CBDs can be reproducibly purified after expression in *E. coli* or other protein production platforms, thus avoiding the use of animals, immunization, and collection of and purification of sera, which is required of polyclonal and monoclonal antibody generation. This ease of manipulation also allows protein fusions to use fluorescent proteins as an alternative to conjugation to fluorescent dyes. However, given the broad binding characteristics of the lactobacilli phage lysin CBDs described here, reagents with improved strain-specific binding specificity would likely be required to test complex multi-strain products. Some alternative approaches include antibody fragments, such as camelid nanobodies and Fabs, which exhibit a narrow binding specificity and can be manipulated and purified in laboratory settings after being isolated from sera [35,36]. In this study, we demonstrated that CBDs fused to fluorescent proteins of different colors can enable the enumeration of viable cells of two lactobacilli in a mixture. This proof-of-concept experiment shows that these proteins may be suitable for determining the potency of LBPs using flow cytometry and supports the further investigation of these proteins with lyophilized LBP preparations. A limitation of this mock potency assay was that it was performed with cells grown in a laboratory culture, and thereby does not include the lyoprotectants and other excipients that are commonly found in LBPs and that may interfere with CBD binding. Future work will build on this study by using lyophilized LBP powders in mock potency assays to determine how they affect CBD binding and by increasing the complexity of the bacterial mixtures. Additionally, future work will investigate other fluorescent binding reagents, for example, nanobodies, phage receptor binding proteins, and other phage proteins with specific binding properties.

Previous studies on lactobacilli phage lysin activity have mostly been limited to the assessments of full lysin protein activity against autoclaved cells or cell wall fragments [25,37,38,39,40]. The contribution of each of the lactobacilli endolysin functional domains to lytic activity remains relatively unexplored. In more well-studied Gram-positive phage endolysins, the cytolytic activity has been found, in some cases, to be independent of or reduced by the CBD, while in other cases, the CBD is required for activity. For example, the cytolytic activity of *C. difficile* phage endolysins CD16/50L and CD27L do not require the CBD for activity, and the presence of the CBD reduces the lytic activity [9,41]. On the other hand, the CBD is required for the activity of *L. monocytogenes* phage endolysins Ply100 and Ply500 [32]. In this study, the activities of three lactobacilli phage endolysins deriving from *L. paracasei* phage CL1, *L. gasseri* phage Jlb1, and *L. delbrueckii* subsp. *bulgaricus* phage ΦJB and their respective EAD domains were characterized by the digestion of autoclaved cell preparations and by the lysis of live cells to evaluate their potential for future development as LBP purity testing reagents. Our results show that the lysins and their EADs exhibited a broad digestive activity of autoclaved LAB cells but a relatively limited lytic activity against live cells. The discrepancy between digestive activity and killing can be attributed to the presence, in live cells, of cell surface polymers, and protein structures, such as S-layers, which are known to interfere with endolysin and autolysin activity [9,42]. This also indicates that digestive activity is not always indicative of lytic and killing activity, which is an important consideration if downstream applications require the killing of live cells. We found that ΦJB-CBD and Jlb1-CBD increased the stability of the full-length lysins against pH extremes and, in Jlb1, increased stability to changes in NaCl concentrations. We also observed that they contributed differentially to the lytic activity of their respective lysins. CL1-CBD was required for optimal activity against the strains we tested, and Jlb1-CBD was required for lytic activity against some strains (e.g., *L. gasseri* SV-16A-US) but not others (e.g., *L. delbrueckii* 15808), and ΦJB-CBD slightly reduced lytic activity. In addition to slightly reducing lytic activity, ΦJB-CBD was found to reduce the diffusion of ΦJB-lys in digestion and soft agar overlay assays and to contribute to the attachment of ΦJB-lys to cell wall debris. Consistent with these results was our finding that ΦJB-CBD binds a surface polymer and not PG. The data presented here will inform decisions about which lysins and EADs to characterize for future assay development with lyophilized LBP preparations. Future work will focus on evaluating lysin lytic activity against lyophilized LBP preparations to determine the influence of lyophilization and product excipients on lysin lytic activity.

The specific goal of demonstrating the microbial purity of LBP products is to demonstrate that the product is relatively free of extraneous organisms, especially pathogenic bacteria. A significant challenge in developing suitable purity assays is that the presence of large numbers of product organisms can obscure the detection or enumeration of contaminating organisms. The ideal LBP purity reagent would possess a potent killing activity that is limited to the product organism(s) so that it can reduce the product organism numbers enough to reveal contaminants by simple, unbiased culture methods. In this study, we showed that incubation with CL1-lys, jlb1-lys, jlb1-EAD, ΦJB-lys, and ΦJB-EAD significantly reduced lab-cultured *L. delbrueckii* populations at the concentrations tested and found that most of these proteins are active in similar buffer conditions. Future work will include expanding the strains tested to those of lyophilized lactobacilli product preparations, pathogenic microorganisms of concern in LBP manufacturing, and testing the effectiveness of these lysins and EADs alone and in combination as purity assay reagents in mock purity assays with lyophilized LBP preparations.

## Figures and Tables

**Table 1:** Endolysin CBD binding screen.

	CL1		Lj965		Jlb1		LL-H		ΦJB	
	% Pos *	Bind	% Pos *	Bind	% Pos *	Bind	% Pos *	Bind	% Pos *	Bind
***L. gasseri***										
19992	98.7 (±1.1)	+	99.6 (±0.1)	+	99.1 (±0.8)	+	65.9 (±6.1)	+	61.5 (±12.8)	+
9857	98.4 (±2.1)	+	99.9 (±0.1)	+	95.9 (±5.8)	+	96.5 (±1.5)	+	98.9 (±1.1)	+
SJ-9E-US	99.3 (±0.77)	+	99.8 (±0.1)	+	99.8 (±0.1)	+	9.9 (±11.4)	−	5.5 (±3.0)	−
SV-16A-US	99.6 (±.42)	+	99.8 (±0.1)	+	99.7 (±0.1)	+	71.6 (±6.1)	+	70.1 (±3.5)	+
***L. jensenii***										
115-3-CHN	69.8 (±0.1)	+	99.9 (±0.1)	+	99.7 (±0.1)	+	88.1 (±16.5)	+	99.4 (±.35)	+
208-1	2.7 (±3.7)	−	99.9 (±0.1)	+	99.8 (±0.1)	+	96.5 (±3.8)	+	99.7 (±0.1)	+
SJ-7A-US	98.2 (±1.3)	+	99.7 (±0.3)	+	99.4 (±0.1)	+	83.9 (±9.2)	+	90.2 (±10.3)	+
***L. crispatus***										
125-2-CHN	22 (±1.2)	−	22.2 (±14.5)	−	38.9 (±16.9)	−	58.3 (±12.3)	+	53.8 (±18.2)	+
33197	79.7 (±2.9)	+	89.6 (±9.5)	+	87.6 (±0.1)	+	88.4 (±14.3)	+	97 (±0.8)	+
53545	97.7 (±2.8)	+	88.5 (±6.9)	+	17.5 (±8.2)	−	96.9 (±2.3)	+	97.3 (±2.9)	+
MV-1A-US	21.1 (±8.8)	−	78.7 (±15.3)	+	15.5 (±5.0)	−	94.5 (±6.8)	+	98 (±1.7)	+
***L. plantarum***										
8014	20.8 (±10.5)	−	20 (±2.1)	−	15.9 (±17.8)	−	2.1 (±0.2)	−	8.1 (±6.3)	−
V	86.8 (±3.4)	+	90.7 (±8.6)	+	63.3 (±9.3)	+	7.5 (±7.9)	−	12.6 (±11.3)	−
***L. vaginalis***										
49540	90.6 (±12.6)	+	95.7 (±4.5)	+	60.0 (±7.1)	+	90 (±12.7)	+	86.2 (±18.1)	+
***L. casei***										
393	98.2 (±2.3)	+	99.3 (±0.1)	+	96.7 (±1.8)	+	10.6 (±9.8)	−	7.54 (±3.8)	−
***L. rhamnosus***										
GG	99.9 (±0.1)	+	99.8 (0.28)	+	99.8 (±0.2)	+	9.1 (±6.3)	−	8.55 (±2.5)	−
LMS2-1	91.2 (± 12)		96.1 (±4.9)	+	96.9 (±4.0)	+	26.4 (±0.1)	−	31 (±0.3)	−
***L. reuteri***										
53608	5.7 (±5.6)	−	8.13 (±7.6)	−	5.1 (±4.7)	−	10.8 (±12.8)	−	15.1 (±13.1)	−
BAA-2837	99.3 (±.2)	+	96.2 (±4.2)	+	60.7 (±11)	+	92.9 (±7.9)	+	95.7 (±1.6)	+
CF-48-3A	1.0 (±0.9)	−	86.7 (±10.2)	+	71.5 (±12.3)	+	19 (±10.1)	−	11.0 (±4.2)	−
***L. acidophilus***										
C	2.0 (±2.6)	−	4.9 (±6.4)	−	0.67 (±0.6)	−	3.45 (±1.4)	−	9.9 (±6.5)	−
V	8.7 (±10.6)	−	6.5 (±6.9)	−	1.1 (±0.4)	−	82.2 (±8.6)	+	84.9 (±10.9)	+
***L. delbrueckii***										
15808	92.4 (±3.7)	+	98.2 (±1.9)	+	95.6 (±4.7)	+	99.8 (±.4)	+	99.9 (±0.1)	+
***L. johnsonii***										
11506	9.0 (±9.2)	−	7.9 (±2.6)	−	3.2 (±2.5)	−	0.88 (±0.8)	−	1.2 (±0.5)	−
135-1-CHN	68.8 (±6.8)	+	84.9 (±4.5)	+	58.7 (±1.3)	+	21.6 (±5.1)	−	27.3 (±5.3)	−
***Lactococcus lactis***										
11454	99.4 (±0.5)	+	99.5 (±0.7)	+	99.7 (±0.5)	+	13.9 (±2.3)	−	20.2 (±5.6)	−
***Enterococcus durans***										
RM86	99.5 (±0.5)	+	99.0 (±0.6)	+	94.2 (±7.2)	+	6.6 (±8.6)	−	3.4 (±2.7)	−
**Total**	17/27		21/27		19/27		14/27		14/27	
**% Bound**	63		78		70		52		52	

* The mean ± SD (*n* = 2) of the percentage of events that have a higher fluorescence intensity than the RFP control. + indicates a >50% of the events exhibited a positive shift in fluorescence intensity compared to controls. − indicates no shift in fluorescence intensity compared to the control.

**Table 2:** Summary of cell wall digestion screen.

	*L. crispatus* 33197	*L. plantarum* 8014	*L. gasseri* SJ-9E-US	*L. delbrueckii* 15808	*L. jensenii* SJ-7A-US
Jlb1-lys ^a^	+	+	+	+	+
Jlb1-EAD ^a^	+	+	+	+	+
Jlb1-CBD ^b^	+, 464	−, 21.8	+, 597	+, 853	+, 4711
CL1-lys ^a^	+	+	+	+	+
CL1-EAD ^a^	+	+	+	+	+
CL1-CBD ^b^	+, 420	−, 108	+, 2534	+, 798	+, 7446
ΦJB-lys ^a^	+	+	+	+	+
ΦJB-EAD ^a^	+	+	+	+	+
ΦJB-CBD ^b^	+, 427	−, 2.57	+, 5.13	+, 3386	+, 817

^a^ Lysin and EAD: + indicates the presence of a digestion zone on autoclaved cell preparation and − indicates lack of zone. ^b^ CBD median fluorescence intensity acquired in CBD binding screen: + indicates increased fluorescence intensity compared to control and − indicates no difference from control.

**Table 3:** Lytic spectrum of lysins and EADs.

	*L. crispatus* 33197	*L. gasseri* SV-16A-US	*L. delbrueckii* 15808	*L. jensenii* SJ-7A-US	*E. durans* RM86
Jlb1-lys ^a^	−	+++	+++	+++	−
Jlb1-EAD ^a^	−	+	+++	+	−
Jlb1-CBD ^b^	+, 464	+, 2138	+, 853	+, 4711	+, 366
CL1-lys ^a^	−	++	+++	+++	−
CL1-EAD ^a^	−	+	++	+	−
CL1-CBD ^b^	+, 420	+, 5070	+, 798	+, 7446	+, 549
ΦJB-lys ^a^	−	+	+++	−	−
ΦJB-EAD ^a^	−	+	+++	+	−
ΦJB-CBD ^b^	+, 427	+, 363	+, 3386	+, 817	−, 3.85

^a^ +++, 50% reduction of OD_600_ within 30 min; ++, 50% reduction of OD_600_ within 60 min; +, <50% reduction of OD_600_ after 60 min; and −, no observable differences as compared to the control. ^b^ Median fluorescence intensity acquired in CBD binding screen.

## Data Availability

Most of the data presented in this study are presented in their entirety in the paper and supplementary material. Other data are available upon reasonable request from the corresponding author.

## Supplementary Materials

The following supporting information can be downloaded at: https://www.mdpi.com/article/10.3390/v15101986/s1, Figure S1: SDS-PAGE gels showing purified lysins, CBDs, and EADs.; Figure S2: Use of Sytox Blue Live/Dead stain to distinguish live from dead cells; Table S1: List of bacterial strain and plasmids used in this study.

## References

[1] Dreher-Lesnick S.M., Stibitz S., Carlson J.J., Paul E. (2017). US regulatory considerations for development of live biotherapeutic products as drugs. Microbiol. Spectr..

[2] (2016). Guidance for Industry: Early Clinical Trials with Live Biotherapeutic Products: Chemistry, Manufacturing, and Control Information. https://www.fda.gov/media/82945/download.

[3] Dreher-Lesnick S.M., Schreier J.E., Stibitz S. (2015). Development of phage lysin LysA2 for use in improved purity assays for live biotherapeutic products. Viruses.

[4] Fernandes S., São-José C. (2018). Enzymes and mechanisms employed by tailed bacteriophages to breach the bacterial cell barriers. Viruses.

[5] Santos S.B., Costa A.R., Carvalho C., Nóbrega F.L., Azeredo J. (2018). Exploiting bacteriophage proteomes: The hidden biotechnological potential. Trends Biotechnol..

[6] Oliveira H., Melo L.D., Santos S.B., Nóbrega F.L., Ferreira E.C., Cerca N., Azeredo J., Kluskens L.D. (2013). Molecular aspects and comparative genomics of bacteriophage endolysins. J. Virol..

[7] Heselpoth R.D., Swift S.M., Linden S.B., Mitchell M.S., Nelson D.C. (2021). Enzybiotics: Endolysins and bacteriocins. Bacteriophages Biol. Technol. Ther..

[8] Broendum S.S., Buckle A.M., McGowan S. (2018). Catalytic diversity and cell wall binding repeats in the phage-encoded endolysins. Mol. Microbiol..

[9] Phothichaisri W., Chankhamhaengdecha S., Janvilisri T., Nuadthaisong J., Phetruen T., Fagan R.P., Chanarat S. (2022). Potential role of the host-derived cell-wall binding domain of endolysin CD16/50L as a molecular anchor in preservation of uninfected *Clostridioides difficile* for new rounds of phage infection. Microbiol. Spectr..

[10] Gómez-Torres N., Dunne M., Garde S., Meijers R., Narbad A., Avila M., Mayer M.J. (2018). Development of a specific fluorescent phage endolysin for in situ detection of *Clostridium* species associated with cheese spoilage. Microb. Biotechnol..

[11] Kong M., Shin J.H., Heu S., Park J.-K., Ryu S. (2017). Lateral flow assay-based bacterial detection using engineered cell wall binding domains of a phage endolysin. Biosensors Bioelectron..

[12] Costa S.P., Dias N.M., Melo L.D., Azeredo J., Santos S.B., Carvalho C.M. (2020). A novel flow cytometry assay based on bacteriophage-derived proteins for *Staphylococcus* detection in blood. Sci. Rep..

[13] Guo T., Xin Y., Zhang C., Ouyang X., Kong J. (2016). The potential of the endolysin Lysdb from *Lactobacillus delbrueckii* phage for combating *Staphylococcus aureus* during cheese manufacture from raw milk. Appl. Microbiol. Biotechnol..

[14] Schmelcher M., Shabarova T., Eugster M.R., Eichenseher F., Tchang V.S., Banz M., Loessner M.J. (2010). Rapid multiplex detection and differentiation of *Listeria* cells by use of fluorescent phage endolysin cell wall binding domains. Appl. Environ. Microbiol..

[15] Roach D.R., Khatibi P.A., Bischoff K.M., Hughes S.R., Donovan D.M. (2013). Bacteriophage-encoded lytic enzymes control growth of contaminating *Lactobacillus* found in fuel ethanol fermentations. Biotechnol. Biofuels.

[16] Lu S., Wang J., Chitsaz F., Derbyshire M.K., Geer R.C., Gonzales N.R., Gwadz M., Hurwitz D.I., Marchler G.H., Song J.S. (2020). CDD/SPARCLE: The conserved domain database in 2020. Nucleic Acids Res..

[17] Gabler F., Nam S.Z., Till S., Mirdita M., Steinegger M., Söding J., Lupas A.N., Alva V. (2020). Protein sequence analysis using the MPI bioinformatics toolkit. Curr. Protoc. Bioinform..

[18] Plaut R.D., Stibitz S. (2015). Improvements to a markerless allelic exchange system for *Bacillus anthracis*. PLoS ONE.

[19] Plaut R.D., Kelly V.K., Lee G.M., Stibitz S., Merkel T.J. (2012). Dissemination bottleneck in a murine model of inhalational anthrax. Infect. Immun..

[20] Nelson D.C., Schmelcher M., Rodriguez-Rubio L., Klumpp J., Pritchard D.G., Dong S., Donovan D.M. (2012). Endolysins as antimicrobials. Adv. Virus Res..

[21] Son B., Kong M., Lee Y., Ryu S. (2021). Development of a novel chimeric endolysin, Lys109 with enhanced lytic activity against *Staphylococcus aureus*. Front. Microbiol..

[22] Hu S., Kong J., Kong W., Guo T., Ji M. (2010). Characterization of a novel LysM domain from *Lactobacillus fermentum* bacteriophage endolysin and its use as an anchor to display heterologous proteins on the surfaces of lactic acid bacteria. Appl. Environ. Microbiol..

[23] Steen A., Buist G., Leenhouts K.J., El Khattabi M., Grijpstra F., Zomer A.L., Venema G., Kuipers O.P., Kok J. (2003). Cell wall attachment of a widely distributed peptidoglycan binding domain is hindered by cell wall constituents. J. Biol. Chem..

[24] Bosma T., Kanninga R., Neef J., Audouy S.A., van Roosmalen M.L., Steen A., Buist G., Kok J., Kuipers O.P., Robillard G. (2006). Novel surface display system for proteins on non-genetically modified gram-positive bacteria. Appl. Environ. Microbiol..

[25] Regulski K., Courtin P., Kulakauskas S., Chapot-Chartier M.-P. (2013). A novel type of peptidoglycan-binding domain highly specific for amidated D-Asp cross-bridge, identified in *Lactobacillus casei* bacteriophage endolysins. J. Biol. Chem..

[26] Antikainen J., Anton L., Sillanpää J., Korhonen T.K. (2002). Domains in the S-layer protein CbsA of *Lactobacillus crispatus* involved in adherence to collagens, laminin and lipoteichoic acids and in self-assembly. Mol. Microbiol..

[27] Fina Martin J., Palomino M.M., Cutine A.M., Modenutti C.P., Fernández Do Porto D.A., Allievi M.C., Zanini S.H., Mariño K.V., Barquero A.A., Ruzal S.M. (2019). Exploring lectin-like activity of the S-layer protein of *Lactobacillus acidophilus* ATCC 4356. Appl. Microbiol. Biotechnol..

[28] Pei Z., Sadiq F.A., Han X., Zhao J., Zhang H., Ross R.P., Lu W., Chen W. (2021). Comprehensive scanning of prophages in *Lactobacillus*: Distribution, diversity, antibiotic resistance genes, and linkages with CRISPR-Cas systems. Msystems.

[29] Buist G., Steen A., Kok J., Kuipers O.P. (2008). LysM, a widely distributed protein motif for binding to (peptido) glycans. Mol. Microbiol..

[30] Lee K.O., Kong M., Kim I., Bai J., Cha S., Kim B., Ryu K.-S., Ryu S., Suh J.-Y. (2019). Structural Basis for Cell-Wall Recognition by Bacteriophage PBC5 Endolysin. Structure.

[31] Gründling A., Schneewind O. (2006). Cross-linked peptidoglycan mediates lysostaphin binding to the cell wall envelope of *Staphylococcus aureus*. J. Bacteriol..

[32] Loessner M.J., Kramer K., Ebel F., Scherer S. (2002). C-terminal domains of *Listeria monocytogenes* bacteriophage murein hydrolases determine specific recognition and high-affinity binding to bacterial cell wall carbohydrates. Mol. Microbiol..

[33] Eugster M.R., Haug M.C., Huwiler S.G., Loessner M.J. (2011). The cell wall binding domain of *Listeria* bacteriophage endolysin PlyP35 recognizes terminal GlcNAc residues in cell wall teichoic acid. Mol. Microbiol..

[34] Chiron C., Tompkins T.A., Burguière P. (2018). Flow cytometry: A versatile technology for specific quantification and viability assessment of micro-organisms in multistrain probiotic products. J. Appl. Microbiol..

[35] Teillaud J.-L. (2012). From whole monoclonal antibodies to single domain antibodies: Think small. Single Domain Antibodies.

[36] Brinkmann U., Kontermann R.E. (2017). The Making of Bispecific Antibodies.

[37] Deutsch S.-M., Guezenec S., Piot M., Foster S., Lortal S. (2004). Mur-LH, the broad-spectrum endolysin of *Lactobacillus* helveticus temperate bacteriophage Φ-0303. Appl. Environ. Microbiol..

[38] Vasala A., Välkkilä M., Caldentey J., Alatossava T. (1995). Genetic and biochemical characterization of the *Lactobacillus delbrueckii* subsp. lactis bacteriophage LL-H lysin. Appl. Environ. Microbiol..

[39] Sugahara K., Yokoi K.-j., Nakamura Y., Nishino T., Yamakawa A., Taketo A., Kodaira K.-I. (2007). Mutational and biochemical analyses of the endolysin LysgaY encoded by the *Lactobacillus gasseri* JCM 1131T phage φgaY. Gene.

[40] Ribelles P., Rodríguez I., Suárez J.E. (2012). LysA2, the *Lactobacillus casei* bacteriophage A2 lysin is an endopeptidase active on a wide spectrum of lactic acid bacteria. Appl. Microbiol. Biotechnol..

[41] Wang Q., Euler C.W., Delaune A., Fischetti V.A. (2015). Using a novel lysin to help control *Clostridium difficile* infections. Antimicrob. Agents Chemother..

[42] Martínez B., Rodríguez A., Kulakauskas S., Chapot-Chartier M.-P. (2020). Cell wall homeostasis in lactic acid bacteria: Threats and defences. FEMS Microbiol. Rev..

