# Supplementary material for: Characterization of Lactobacilli Phage Endolysins and Their Functional Domains–Potential Live Biotherapeutic Testing Reagents
Source: Viruses. 2023 Sep 23;15(10):1986. doi: 10.3390/v15101986 (PMC10610939; doi:10.3390/v15101986)
Supplement: Supplementary file 1 [file viruses-15-01986-s001.zip › viruses-2590349-supplementary.pdf]

Characterization of Lactobacilli Phage Endolysins and Their Functional Domains – Potential Reagents for Live Biotherapeutic Product Microbiological Testing

Robert J. Dorosky[a]*, Stephanie L. Lola[a], Haleigh A. Brown[a], Jeremy E. Schreier[b], Sheila M. Dreher-Lesnick[a], and Scott Stibitz[a]

[a]Office of Vaccines Research and Review, Division of Bacterial, Parasitic and Allergenic Products, Center for Biologics Evaluation and Research, U.S. Food and Drug Administration, Silver Spring, MD 20993, USA

[b]Department of Marine Sciences, University of Georgia, Athens, GA 30602, USA

* Correspondence

Supplemental Figures

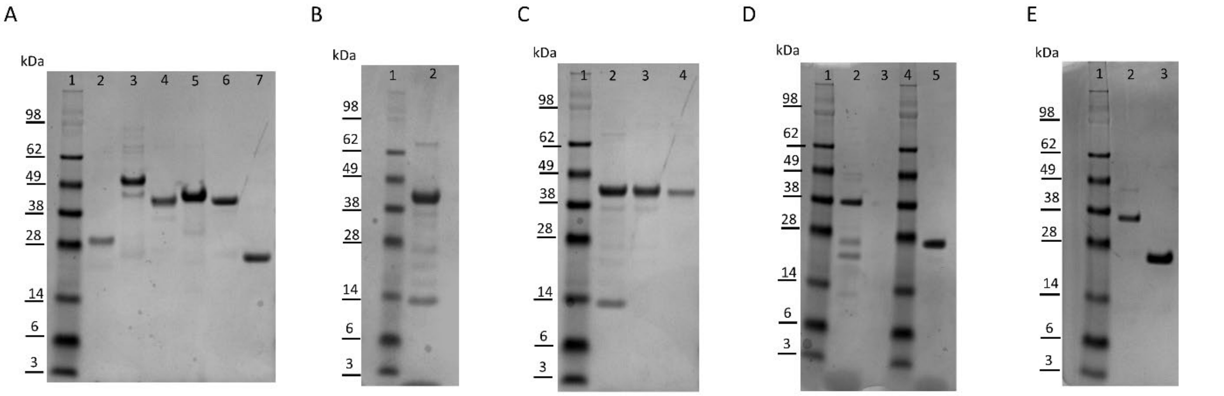

Lane	Protein	kDa
2	TurboRFP	28.4
3	TurboRFP-CL1-CBD	48.8
4	TurboRFP-LL-H-CBD	39.1
5	TurboGFP-LL-H-CBD	39.1
6	CL1-lys	38.7
7	ϕJB-EAD	23.8

Lane	Protein	kDa
2	TagRFP-Jlb1-CBD	41.2

Lane	Protein	kDa
2	TurboRFP-Jlb1-CBD	40.6
3	TurboRFP-ϕJB-CBD	38.8
4	TurboRFP-Lj965-CBD	40.5

Lane	Protein	kDa
2	Jlb1-lys	34.9
3	Empty	n/a
4	Marker	n/a
5	Jlb1-EAD	24.2

Lane	Protein	kDa
2	ϕJB-lys	33.2
3	CL1-EAD	22.9

Figure S1. SDS PAGE gels showing purified lysins, CBDs, and EADs. Proteins were loaded on NupAGE 4-12% BisTris gels and stained with Coomasie Blue. The expected molecular mass of each protein and the lane in which the purified protein was run in indicated in the boxes below the gels. Molecular weight markers are identified on the left. Please note that under boiling SDS conditions TurboRFP and TagRFP is know to fragment.

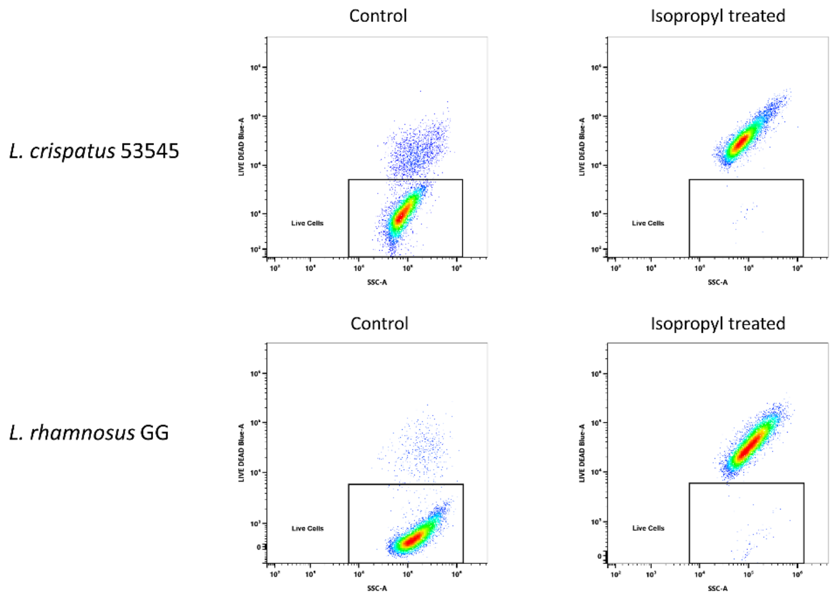

Figure S2. Use of SYTOX Blue Live/Dead stain to distinguish live from dead cells. SYTOX blue (ThermoFisher) is a cell impermeant nucleic acid stain that enters cells with damaged membranes and binds nucleic acids. (Top) untreated *L. crispatus* 53545 control (Left) and (Right) isopropyl alcohol treated cells (70%, 5 min). (Bottom) Untreated *L. rhamnosus* GG control (Left) and (Right) isopropyl alcohol treated cells.

Table S1. List of bacterial strains and plasmids used in this study

**Bacterial Strains**	**Description**	**Source**
Lactobacillus gasseri 19992		ATCC
Lactobacillus gasseri 9857		ATCC
Lactobacillus gasseri SJ-9E-US		BEI
Lactobacillus gasseri SV-16A-US		BEI
Lactobacillus jensenii 115-3-CHN		BEI
Lactobacillus jensenii 208-1		BEI
Lactobacillus jensenii SJ-7A-US		BEI
Lactobacillus crispatus 125-2-CHN		BEI
Lactobacillus crispatus 33197		ATCC
Lactobacillus crispatus 53545		ATCC
Lactobacillus crispatus MV-1A-US		BEI
Lactiplantibacillus plantarum 8014		ATCC
Lactiplantibacillus plantarum V	Product isolate	[1]
Limosilactobacillus vaginalis 49540		ATCC
Lacticaseibacillus casei 393		ATCC

Enterococcus durans RM62	Breast milk isolate, Identified by MALDI-TOF	This Study
Limosilactobacillus reuteri 53608		ATCC
Limosilactobacillus reuteri BAA-2837		ATCC
Limosilactobacillus reuteri CF-48-3A		BEI
Lacticaseibacillus rhamnosus GG		ATCC
Lacticaseibacillus rhamnosus LMS2-1		BEI
Lactobacillus acidophilus C	Product Isolate	[1]
Lactobacillus acidophilus V	Product Isolate	[1]
Lactobacillus delbrueckii sub lactis 15808		ATCC
Lactococcus lactis 11454		ATCC
Lactobacillus johnsonii 11506		ATCC
Lactobacillus johnsonii 135-1-CHN		BEI
Escherichia coli BL21		Novagen
**Plasmids**	**Description**	**Source/ Accession Number**
pSDL221	pET22b(+) containing 693bp fragment encoding TurboRFP cloned into XbaI and SacI	This study / OR372164
pSDL218	pET22b(+) containing 711bp fragment encoding TurboGFP cloned into XbaI and SacI	This study / OR372165
pRJD193	pET22b(+) containing 1298bp fragment that includes TurboRFP CL1 CBD gene fusion (AA 155-350)	This study / OR372166
pRJD198	pET22b(+) containing 1064bp fragment that includes TurboRFP jlb1 CBD gene fusion (AA 192-310)	This study / OR372167
pSLL166	pET22b(+) containing 1315bp fragment that includes TagRFP jlb1 CBD gene fusion (AA 192-310)	This study / OR372168
pRJD209	pET22b(+) containing 1052bp fragment that includes TurboRFP Lj965 CBD gene fusion (196-310)	This study / OR372169
pRJD201	pET22b(+) containing 1022bp fragment that includes TurboRFP LLh CBD gene fusion (AA 194-298)	This study / OR372170
pSLL160	pET22b(+) containing 1039bp fragment that includes TurboGFP LLh CBD gene fusion (AA 194-298)	This study / OR372172
pRJD202	pET22b(+) containing 1016bp fragment that includes TurboRFP phiJB CBD gene fusion (AA 192-294)	This study / OR372171
pRJD488	pET22b(+) containing 1050bp fragment that includes CL1 lysin coding sequence	This study / OR372173
pRJD489	pET22b(+) containing 629bp fragment including the CL1 EAD coding sequence (AA 1-205)	This study / OR372174

pRJD492	pET22b(+) containing 930bp fragment that includes Jlb1 lysin coding sequence	This study / OR372175
pRJD493	pET22b(+) containing 654bp fragment including the Jlb1 EAD coding sequence (AA 1-212)	This study / OR372176
pRJD490	pET22b(+) containing 882bp fragment that includes PhiJB lysin coding sequence	This study / OR372177
pRJD491	pET22b(+) containing 645bp fragment including the phiJB EAD coding sequence (AA 1-209)	This study / OR372178

References

1. Dreher-Lesnick, S. M.; Schreier, J. E.; Stibitz, S., Development of phage lysin LysA2 for use in improved purity assays for live biotherapeutic products. *Viruses* **2015,** 7, (12), 6675-6688.
